# Supplementary material for: Biosocial correlates of adolescent’s knowledge on pubertal changes in rural Bangladesh: A structural equation model
Source: PLoS One. 2022 Mar 22;17(3):e0264515. doi: 10.1371/journal.pone.0264515 (PMC8939805; doi:10.1371/journal.pone.0264515)
Supplement: Attachment — Submitted filename: 4.PONE-D-21-15883_reviewer.pdf [file pone.0264515.s001.pdf]

PLOS ONE

Impact of School-based Sexual and Reproductive Health Education among Secondary Student's Knowledge on Pubertal Changes in Rural Bangladesh

--Manuscript Draft--

**Manuscript Number:**	PONE-D-21-15883
**Article Type:**	Research Article
**Full Title:**	Impact of School-based Sexual and Reproductive Health Education among Secondary Student's Knowledge on Pubertal Changes in Rural Bangladesh
**Short Title:**	Impact of Sexual and Reproductive Health Education among Student's Knowledge on Pubertal Changes in Bangladesh
**Corresponding Author:**	Iqramul Haq<br>Sher-e-Bangla Agricultural University<br>Dhaka, --- Select One --- BANGLADESH
**Keywords:**	Knowledge of puberty, adolescent, behavioural control, Structural equation model
**Abstract:**	Introduction<br>Adolescence is the critical stage when an individual shifts from childhood to adulthood with accelerated hormonal, emotional, social, and physiological changes. The present study aimed to identify factors that are associated with puberty knowledge among school-going rural adolescents in Bangladesh.<br>Materials and Methods<br>This cross-sectional study was conducted on 2724 school-going (grades 6-9) adolescents aged 10-18 years who resided only in rural areas of Bangladesh. We applied the structural equation model to explore factors related to the knowledge of pubertal changes among rural adolescents in Bangladesh.<br>Results<br>Out of the 2724 adolescents, 51% were male and 49% were female in this analysis. The mean age of the adolescents and their years of schooling was 14.67 and 8.08 , respectively. The structural equation model result showed a significant association among gender (p <0.001), education (p <0.001), age (p <0.001), and parental limit setting (p <0.001) with student's knowledge on pubertal changes. From the result of the simultaneous equation model, sex, age, years of schooling, Academic performance, teacher concern were significant factors that directly influencing adolescent knowledge on puberty while mother having no education, parental limit-setting, peer connection, and peer regulation were influencing adolescent knowledge on puberty both direct and indirect ways.<br>Conclusion<br>This study provides insights into how school-based sexual health education can be made more effective to increase adolescent's knowledge about pubertal changes in rural Bangladesh.
**Order of Authors:**	Md Injamul Haq Methun
	M. Sheikh Giash Uddin
	Md. Ismail Hossain
	Md. Jakaria Habib
	Ahmed Abdus Saleh Saleheen
	Iqramul Haq
**Additional Information:**	
**Question**	**Response**
**Financial Disclosure**<br><br>Enter a financial disclosure statement that describes the sources of funding for the	The author(s) received no specific funding for this work.

work included in this submission. Review the submission guidelines for detailed requirements. View published research articles from *PLOS ONE* for specific examples.<br><br>This statement is required for submission and **will appear in the published article** if the submission is accepted. Please make sure it is accurate.<br><br>**Unfunded studies**<br>Enter: *The author(s) received no specific funding for this work.*<br><br>**Funded studies**<br>Enter a statement with the following details:<br>• Initials of the authors who received each award<br>• Grant numbers awarded to each author<br>• The full name of each funder<br>• URL of each funder website<br>• Did the sponsors or funders play any role in the study design, data collection and analysis, decision to publish, or preparation of the manuscript?<br>• **NO** - Include this sentence at the end of your statement: *The funders had no role in study design, data collection and analysis, decision to publish, or preparation of the manuscript.*<br>• **YES** - Specify the role(s) played.<br><br>* typeset	
**Competing Interests**<br><br>Use the instructions below to enter a competing interest statement for this submission. On behalf of all authors, disclose any competing interests that could be perceived to bias this work—acknowledging all financial support and any other relevant financial or non-financial competing interests.<br><br>This statement is required for submission and **will appear in the published article** if the submission is accepted. Please make sure it is accurate and that any funding sources listed in your Funding Information	The authors have declared that no competing interests exist.

later in the submission form are also declared in your Financial Disclosure statement.<br><br>View published research articles from *PLOS ONE* for specific examples.<br><br>**NO authors have competing interests**<br><br>Enter: *The authors have declared that no competing interests exist.*<br><br>**Authors with competing interests**<br><br>Enter competing interest details beginning with this statement:<br><br>*I have read the journal's policy and the authors of this manuscript have the following competing interests: [insert competing interests here]*<br><br>* typeset	
**Ethics Statement**<br><br>Enter an ethics statement for this submission. This statement is required if the study involved:<br><br>• Human participants<br>• Human specimens or tissue<br>• Vertebrate animals or cephalopods<br>• Vertebrate embryos or tissues<br>• Field research<br><br>Write "N/A" if the submission does not require an ethics statement.<br><br>General guidance is provided below. Consult the submission guidelines for detailed instructions. **Make sure that all information entered here is included in the Methods section of the manuscript.**	The study protocol was taken from the Jagannath University, Bangladesh institutional ethical committee (JEC) (Under the project number 37.20.0000.004.033.020.2016.7725).

**Format for specific study types**<br><br>**Human Subject Research (involving human participants and/or tissue)**<br>• Give the name of the institutional review board or ethics committee that approved the study<br>• Include the approval number and/or a statement indicating approval of this research<br>• Indicate the form of consent obtained (written/oral) or the reason that consent was not obtained (e.g. the data were analyzed anonymously)<br><br>**Animal Research (involving vertebrate animals, embryos or tissues)**<br>• Provide the name of the Institutional Animal Care and Use Committee (IACUC) or other relevant ethics board that reviewed the study protocol, and indicate whether they approved this research or granted a formal waiver of ethical approval<br>• Include an approval number if one was obtained<br>• If the study involved *non-human primates*, add *additional details* about animal welfare and steps taken to ameliorate suffering<br>• If anesthesia, euthanasia, or any kind of animal sacrifice is part of the study, include briefly which substances and/or methods were applied<br><br>**Field Research**<br><br>Include the following details if this study involves the collection of plant, animal, or other materials from a natural setting:<br>• Field permit number<br>• Name of the institution or relevant body that granted permission	
**Data Availability**<br><br>Authors are required to make all data underlying the findings described fully available, without restriction, and from the time of publication. PLOS allows rare exceptions to address legal and ethical concerns. See the PLOS Data Policy and FAQ for detailed information.	No - some restrictions will apply

A Data Availability Statement describing where the data can be found is required at submission. Your answers to this question constitute the Data Availability Statement and **will be published in the article**, if accepted.<br><br>**Important:** Stating 'data available on request from the author' is not sufficient. If your data are only available upon request, select 'No' for the first question and explain your exceptional situation in the text box.<br><br>Do the authors confirm that all data underlying the findings described in their manuscript are fully available without restriction?	
**Describe where the data may be found in full sentences. If you are copying our sample text, replace any instances of XXX with the appropriate details.**<br><br>• If the data are **held or will be held in a public repository**, include URLs, accession numbers or DOIs. If this information will only be available after acceptance, indicate this by ticking the box below. For example: *All XXX files are available from the XXX database (accession number(s) XXX, XXX.*).<br>• If the data are all contained **within the manuscript and/or Supporting Information files**, enter the following: *All relevant data are within the manuscript and its Supporting Information files.*<br>• If neither of these applies but you are able to provide **details of access elsewhere**, with or without limitations, please do so. For example:<br><br>*Data cannot be shared publicly because of [XXX]. Data are available from the XXX Institutional Data Access / Ethics Committee (contact via XXX) for researchers who meet the criteria for access to confidential data.*<br><br>*The data underlying the results presented in the study are available from (include the name of the third party*	Data cannot be shared publicly because of the restriction of data authority. However, if you will want data then we must try to add a data source link.

and contact information or URL).<br>• This text is appropriate if the data are owned by a third party and authors do not have permission to share the data.<br><br>* typeset	
Additional data availability information:	

Powered by Editorial Manager® and ProduXion Manager® from Aries Systems Corporation

Manuscript

Click here to access/download;Manuscript;Pubertal Knowledge (Plos One)_WTF.docx

"Impact of School-based Sexual and Reproductive Health Education among Secondary Student's Knowledge on Pubertal Changes in Rural Bangladesh"

Md Injamul Haq Methun[1], M. Sheikh Giash Uddin[2], Md. Ismail Hossain[2], Md. Jakaria Habib[2], Ahmed Abdus Saleh Saleheen[2], Iqramul Haq[3,*]

1 Statistics Discipline, Tejgaon College, Dhaka-1215, Bangladesh

2 Department of Statistics, Jagannath University, Dhaka-1100, Bangladesh

3 Department of Agricultural Statistics, Sher-e-Bangla Agricultural University, Dhaka-1207, Bangladesh

Disclosure statement: No potential conflict of interest was reported by the authors.

Funding: None

*Corresponding Author: Iqramul Haq, Department of Agricultural Statistics, Sher-e-Bangla Agricultural University, Dhaka-1207, Bangladesh

E-mail: iqramul.haq@sau.edu.bd

Impact of School-based Sexual and Reproductive Health Education among Secondary Student's Knowledge on Pubertal Changes in Rural Bangladesh

Background

Adolescence is the critical stage when an individual shifts from childhood to adulthood with accelerated hormonal, emotional, social, and physiological changes. The present study aimed to identify factors that are associated with puberty knowledge among school-going rural adolescents in Bangladesh.

Methods

This cross-sectional study was conducted on 2724 school-going (grades 6-9) adolescents aged 10-18 years who resided only in rural areas of Bangladesh. We applied the structural equation model to explore factors related to the knowledge of pubertal changes among rural adolescents in Bangladesh.

Results

Out of the 2724 adolescents, 51% were male and 49% were female in this analysis. The mean age of the adolescents and their years of schooling was 14.67 ± 1.32 and $8.08 \pm 1.02\ years$, respectively. The structural equation model result showed a significant association among gender ($p<0.001$), education ($p<0.001$), age ($p<0.001$), and parental limit setting ($p<0.001$) with student's knowledge on pubertal changes. From the result of the simultaneous equation model, sex, age, years of schooling, Academic performance, teacher concern were significant factors that directly influencing adolescent knowledge on puberty while mother having no education, parental limit-setting, peer connection, and peer regulation were influencing adolescent knowledge on puberty both direct and indirect ways.

Conclusion

This study provides insights into how school-based sexual health education can be made more effective to increase adolescent's knowledge about pubertal changes in rural Bangladesh.

Keywords: Knowledge of puberty, adolescent, behavioural control, Structural equation model

Introduction

Adolescence is the most complicated and complex period of life with rapid hormonal, emotional, biological, cognitive, and social transformations [1]. To make healthy adults from childhood, it is essential to have correct information about physical and psychological changes they will go through. This critical transition period of life span starts with the onset of puberty

[2]. The onset of puberty causes physical and physiological, psychological, social, mental, cognitive, and behavioural changes [3, 4]. The physical changes during puberty among girls are breast foliage, rapid height and weight rise, pubic and axillary hair development, and hip widening menstrual onset in girls [5-8]. Likewise, boys experience changes in tone, increase in shoulder width, night-time ejaculation, penis enlargement, axillary hair growth, boys' facial hair [9]. At the same time, adolescents go through a profound psychological transformation, and they experience a wide change in their self-image, attitude, relationship. Moreover, they also experience a sudden change in the social, academic, and other environmental influences [10]. Those multidimensional changes make them confused, insecure, and self-centred [11, 12]. As a result, mental disorders, such as severe depression, anxiety disorders, eating disorders, and substance use disorders, can arise during puberty [13]. More than 50 percent of female adolescents had experienced moderate to severe stress due to changes in puberty [2].

If adolescent can recognize themselves and their body functioning during the adolescence period, they may experience a smooth pubertal period and establish a healthy attitude towards sex, marriage, parenthood, and family. Therefore, they need an explanation about the process of puberty that unfolds for everyone and what changes to expect. The lack of adequate knowledge and confusion about their own physical and/or sexual development (i.e., changes in growth) can expose problems for them [1]. Moreover, misconceptions, taboo and myths about sex, sexuality, reproduction, and contraception also deprived them of obtaining correct knowledge [14]. Thus, the fragile teenagers are getting more fragile along with their distinctive development stage.

Society is responsible for ensuring informed puberty so that adolescents can lead healthy, secure, productive, and enjoyable lives and protect themselves from reproductive health problems. But in the socio-cultural context, parents neglect their duty to pass health information to their children because of either shame or indifference or life obligations. Ignoring their child's difficulty and discomfort in this process and putting their responsibility to guide their kids on the shoulders of teachers who may also overlook it [15]. In such instances, adolescents have no choice other than to go to their peers, siblings, and mass media. It might tend to inaccurate or insufficient knowledge and misconception of the condition [16]. In reality, these adolescents can create problems for themselves and their parents, putting their physical, emotional, and social well-being at stake [15].

Both the Government of Bangladesh (GOB) and NGOs have undertaken various activities at different times to alleviate the current disparity between the need and comprehension of adequate knowledge and awareness of puberty. Those activities are

implemented under the adolescent sexual and reproductive health (ASRH) program initiatives. There were 32 adolescent sexual and reproductive health awareness-raising and service delivery initiatives introduced in Bangladesh between 2005 and 2015 [17]. To make awareness raising program successful, it is essential to find out the factors that affect adolescent's knowledge of pubertal changes. No specific research has been done so far on how parents and our society hinder adolescent's knowledge of pubertal changes. However, to make the awareness-raising program effective, it is highly demanded to carry out an empirical study about how family and community level factors effects adolescent's knowledge of pubertal changes.

Therefore, in the situation of minimal evidence, this study set out to add empirical evidence effect of socio-demographic factors and controlling behaviour of our society on adolescent's knowledge of pubertal changes.

Conceptual Framework

In this study, the effect of socio-demographic factors and controlling behaviour was assessed considering Bronfenbrenner's bioecological model [10, 18]

Figure 1. Conceptual Frame work

Methods

Data

Data for this cross-sectional study was carried out by the Department of Statistics of Jagannath University in March 2019 in the rural areas of Bangladesh. Data regarding socio-demographic, psychosocial, and reproductive health knowledge and status-related information were collected from school-going (grades 6-9) adolescents aged 10-18 years in rural areas of Bangladesh.

The survey consisted of two modules of data set named household module and a school module of data sets. Finally, two data modules were merged for analysis. The survey used a three-stage stratified sampling procedure to collect information for school-going students of grade VI to IX. A total of 3000 adolescents aged 10-24 were identified from the selected areas. In the survey, 3014 adolescents were successfully interviewed in rural areas of Bangladesh, with 1504 adolescents were in the household module and 1509 adolescents were in the school module. After deleting the missing and incomplete cases, there were 2724 cases used for the final analysis.

Ethical Consideration

All participants of this study were informed before data collection about the purpose of the study. Verbal consent was taken from the headmasters of all secondary schools and students before data collection. Participant anonymity and confidentiality of data were ensured, and then participants were provided with information about the nature and purpose of the study, the procedure, and the right to withdraw their data from the study. The study protocol was taken from the Jagannath University, Bangladesh institutional ethical committee (JEC) (Under the project number 37.20.0000.004.033.020.2016.7725).

Study population

Outcome Variable

The outcome variable was knowledge of pubertal changes. To assess adolescent's knowledge regarding pubertal changes, students asked about the physical, mental, and behavioural changes during puberty. The number of changes they can correctly identify, which is described on the national curriculum, is marked as the score of their knowledge regarding the pubertal changes. The maximum and minimum scores of knowledges on pubertal changes could be 18 and 1, respectively.

Independent variables

Socio-demographic determinants

Independent variables were year of schooling, age, sex (male and female), religion (Islam and others), Father's education (no education, primary, secondary and higher) and Mother's education (no education, primary, secondary and higher).

Contextual Factors

These contextual factors were measured by utilizing the method from Amoateng and Kalule-Sabiti [19].

Parental behavioural control (PBC). Parental behavioural control is a composite. It measured as a mean score of five questions about how much their parents are conscious about their friends, night stay, and money spending. The responses are taken into a three-point Likert scale categorized as 'Does not know', 'know a little', and 'knows a lot'.

Parental limit settings (PLS). To understand the parental limit-setting behaviour respondents are asked four questions about parenting actions. Which are how much did one of your parents or primary caregivers do the following things over the past 30 days: 'regulate the period of time you could watch televisions', 'inspect whether or not homework was done',

‘help to prepare home assignment, check the exam grade sheet’. The responses are taken on a four-point Likert scale- 1. Never, 2. On rare occasion 3. Occasionally 4. Frequently. The average value of these four questions taken as the child’s parental limit setting.

Community Disorganization (CD). The mean score of five questions related to how often they are disrupted in the community is used to assess the community’s disorganization. The questions are- “How often do you see the litter or trash on the sidewalks and streets?”, “How often do you see graffiti on building walls?”, “How often you do you see alcoholics or drug sales?”, “How often you become afraid or worried when you are walking through the empty place?”, “How frequently burglary happened?”. The responses range from 1 to 3. 1 stands for “Often”, 2 for “Sometimes” and 3 for “Never”.

Community Psychological control (CPC). The average response of three five-point Likert scale questions is used as community psychological control. The problems are related to is your neighbours always watch what you are doing, interfere with what you are doing, and is noisy.

Peer Connection (PeC). The average values of three questions on when and how respondents communicate the friend via ‘phone conversation’ ‘go over to one another residences’ and ‘go together for a movie/skating/shopping/sports event’ is evaluated as connection with friends. The answers are coded as ‘never’, ‘once a month’, ‘once per week’, and ‘many times a week or every day’.

Peer Psychological control (PePC). Psychological control of peers is quantified by the mean score of the answers to three questions on how often their peers disagree with you, how often they make you feel that your ideas are not as good as theirs,' humiliate or bug you' and 'drag you down.' The answers are coded as:' hardly ever,' once every month,' once per week,' a couple of times in a week ', and "every day.

Peer regulation (PeR). Friend’s regulation focuses on friends’ influence in complying with laws and regulations. It is measured as the mean of two questions about how often the friend “supports you do whatever is correct” and “motivates you to abide by the rules. ‘Never’, ‘once a month’, ‘once a week’, ’a few times a week and every day’ are the replies.

Teacher’s concern (TC). The teacher’s concern demonstrates teachers’ supportive, friendly relationship with students that supports student’s academic well-being. To measure teacher concern, four questions asked about teachers’ willingness to assist them with schoolwork and solve personal problems. The average of responses is used as a measure of teacher’s concern.

Student's Academic Performance (SAP). Academic success is assessed on an ordinary scale by a single question:' In general, how well are you doing in school? The replies are: 1 'way above average', 2 'a little above average', 3 'average', 4 'at just below average' and 5 'far below average'.

Statistical Analysis

Simple descriptive analysis and multivariate statistical analysis were performed in this study. The socio-demographic characteristics were presented by frequency and percentage distribution. At the same time, median with maximum and minimum value was used to describe the general information of contextual factors. The generalized structural equation model was also used to test the hypothetical causal path in which individual level, family level and community level variables affect adolescent's knowledge on pubertal changes. There was no latent variable included in the model. All the analysis was conducted using the software R version 3.6.0. A Simultaneous equation model was fitted using "lavaan" package [20].

Results

Socio-demographic Characteristics

Table 1 demonstrates descriptive statistics of socio-demographic variables. There was almost an equal number of representatives of both sexes in the survey data (male 50.81% and female 49.19%). The mean age of adolescents and their years of schooling was 14.67 and 8.08, respectively. More than half of the adolescent's parents (father and mother) had achieved a secondary level education (51.19% for father and 57.27% for mother), whereas only 7.49 percent father had higher education and only a tiny portion mother completed higher education (2.75 %).

Table 1. Descriptive statistics of socio-demographic characteristics.

Variable	Group	Frequency/Mean±SD	Percentage
**Year of Schooling**		8.08±1.02	
**Age**		14.67±1.32	
**Sex**	Male	1384	50.81
	Female	1340	49.19
**Religion**	Islam	2556	93.83
	Others	168	6.17
**Father's education**	No education	500	18.35
	Primary	623	22.87
	Secondary	1397	51.19
	Higher	204	7.49
**Mother's education**	No education	412	15.13
	Primary	677	24.85
	Secondary	1560	57.27
	Higher	75	2.75

Contextual Factors

Descriptive statistics of contextual factors (Table 2) showed that parental behavioural control and community disorganization ranges from 1 to 3, while parental limit setting ranging from 1 to 4 and the upper limit for the remaining variables is 5. Family level variables parental behavioural control and Parental limit setting had median value 2.67 and 2.75 respectively. The median of parental behavioural control was close to maximum value indicates high parental behavioural control for the adolescents. Median value of peer psychological control was 1.25 which was very close to the lower limit of the range indicating adolescents who are merely psychologically controlled by the peer. But the peer regulation had median value 4 and for peer connection was 3.

Table 2: Descriptive statistics of contextual factors

Factors	Median	Minimum	Maximum
Student's Academic performance	0.67	1	5.00
Parental behavioural control	2.67	1	3.00
Parental limit setting	2.75	1	4.00
Peer connection	3.00	1	5.00
Peer psychological control	1.25	1	5.00
Peer Regulation	4.00	1	5.00
Community disorganization	1.67	1	3.00
Community Psychological control	2.67	1	5.00
Teacher's concern	4.00	1	5.00

Generalized structural Equation Model of Knowledge on Pubertal Changes

The structural equation model was over-identified as the degrees of freedom of the model are 73, indicating that the number of free parameters to be estimated is less than the number of distinct values in the sample variance and covariance matrix. The model fit test statistic was with p-value less than 0.001. The Goodness of fit index value (GFI = 0.89) indicated a good fit model. Moreover, Values of error-of-approximation based fit indices (RMSEA = 0.07, RMR = 0.02) was also specified the model as a good fit.

The path coefficient of the structural equation model showed on table-3. The result of structural equation model showed that gender (β = 0.753, P<0.001), education (β = 0.64, P<0.001), age (β = 0.297, P<0.001), and parental limit setting (β = 0.423, P<0.001) had significant effect on student's knowledge on pubertal changes.

Table 3. Result of simultaneous equation model of contextual factors with knowledge about puberty.

Path	Estimate	P-value	Path	Estimate	P-value
KAP ←Gender	0.753	<0.001	EOS ←PLS	-0.053	0.011
KAP ←Education	0.64	<0.001	EOS ←PPC	-0.005	0.889
KAP ←Age	0.297	<0.001	SAP←ME1	0.139	0.001
KAP ←Religion	-0.381	0.10	SAP← ME2	0.108	0.006
KAP ←SAP	-0.413	0.03	SAP← ME3	0.094	0.010

KAP ←PBC	0.051	0.703	SAP ←PBC	-0.054	<0.001
KAP ←PLS	-0.413	<0.001	SAP ←PLS	-0.030	<0.001
KAP ←PEPC	-0.216	0.009	SAP ←PPC	0.006	0.592
KAP ←PEC	-0.351	<0.001	PBC ←PER	0.073	<0.001
KAP ←PER	0.133	0.01	PBC ←PEPC	-0.064	<0.001
KAP ←TC	0.125	0.102	PBC ←CD	-0.064	<0.001
KAP ← ME1	-0.783	0.04	PBC ←TC	0.074	<0.001
KAP ← ME2	-0.452	0.200	PLS ←PEC	0.063	<0.001
KAP ← ME3	-0.256	0.437	PLS ←PER	0.007	0.603
EOS ← ME1	0.078	0.755	PLS ←CD	-0.048	0.052
EOS ← ME2	0.047	0.634	PLS ←CPC	-0.033	0.017
EOS ← ME3	0.104	0.259	PLS ←TC	0.124	<0.001
EOS ←FWS	0.016	0.519	PPC ←PEPC	0.082	<0.001
EOS ←PBC	0.016	0.519	PPC ←CD	0.10	0.561

******EOS = Year of Schooling; ME = Mother's Education; FE = Father's Education***

There were several instances of mediation effect in the model. Student's academic performance act as a mediating variable for PPC→SAP→KAP, PLS→SAP→KAP, and PBC→SAP→KAP. Whereas, student's educational aspiration was the mediator for the indirect effect of the father's education (FE→SEA→KAP), mother's education (ME→SEA→KAP), and parental behavioural control (PBC→SEA→KAP) on knowledge about pubertal changes. Community-level variable, community disorganization showed an indirect impact on knowledge about pubertal changes through the mediating variable parental limit setting (CD→PLS→KAP). Adolescents' peer connection, regulation, and psychological control also demonstrated secondary effects on knowledge about pubertal changes through the mediating variables of parental limit settings and parental psychological control (PEPC→PLS→KAP, PER→PLS→KAP, and PEPE→PBC→KAP).

Direct effects, indirect effects, and total effect on Knowledge on pubertal changes

Table 4 showed that sex, age, years of schooling and academic performances significantly influenced adolescent's knowledge of pubertal changes. The sex (β=0.754, $p<0.001$) of adolescents directly affected adolescents' understanding of puberty, which means that female adolescents have a higher understanding of puberty than their male counterparts. Adolescent's

age (β=0.29, p<0.001) had a significant positive impact on knowledge about pubertal changes (Table 5). The knowledge about pubertal changes increased by 0.29 for every year increase in the adolescents' age. Adolescent's year of schooling (β=0.578, p<0.001) also showed a direct positive effect on their knowledge of pubertal changes. This means that as the adolescents spend one year more time at school, their knowledge of pubertal changes significantly increased by 0.578. However, student's academic performances (β= - 0.534, p<0.001) had a negative effect on knowledge about pubertal changes, which indicates that adolescents who were good at school had less knowledge on pubertal changes.

At the family level, the mother's education and parental limit-setting directly affected knowledge on pubertal changes. While parental behavioural control possessed an indirect effect on knowledge on pubertal changes. Mothers without any level of education had a direct (β = -0.872, p< 0.05) negative impact on adolescents' puberty knowledge than mother with higher education in this analysis. Similarly, parents' limitation setting on their child was directly (β = -0.398, p< 0.001), impeding the acquisition of the child's knowledge on pubertal changes. Unlike the above two-variable parental behavioural control (β = 0.029, p< 0.05) indirectly enhanced adolescents' knowledge on pubertal changes.

Among the community level variables, peer connection, peer regulation and teacher's concern demonstrated a significant effect on adolescent's knowledge of pubertal changes. Peer psychological control, community disorganization, and community psychological control failed to significantly impact adolescents' knowledge of pubertal changes. Among the significant variables, peer connection (-0.286, p< 0.001) negatively influenced adolescent's knowledge of pubertal changes more strongly than the other two significant variables. However, peer regulation (β = 0.134, p< 0.05) and teacher's concern (0.069, p< 0.05) produces positive impact on adolescent's knowledge on pubertal changes.

Table 4. Direct effect, indirect effect and total effect of bio-social factors on Knowledge on puberty.

Variable	Category	Direct effect (95% CI)	Indirect effect (95% CI)	Total effect (95% CI)
**Sex**	Female	0.754^{***} [0.74, 0.768]	NA	0.754^{***} [0.74,0.768]
	Male	Ref.	Ref.	Ref.
**Age**		0.29^{***}	NA	0.29^{***}

		[0.284, 0.296]		[0.284, 0.296]
**Religion**	Islam	-0.278 [-0.273, -0.283]	NA	-0.278 [-0.273, -0.283]
	Others	Ref.	Ref.	Ref.
**Year of Schooling**		0.578*** [0.567, 0.589]	NA	0.578*** [0.567,0.589]
**Academic performance**		-0.534*** [-0.524, -0.544]	NA	-0.534*** [-0.524, -0.544]
**Mother's education**	No education	-0.872* [-0.855, -0.889]	-0.076 [-0.075, -0.077]	-0.949* [-0.931, -0.967]
	Primary education	-0.483 [-0.474, -0.492]	-0.059 [-0.058, -0.06]	-0.541 [-0.531, -0.551]
	Secondary education	-0.328 [-0.322, -0.334]	-0.052 [-0.051, -0.053]	-0.38 [-0.373, -0.387]
	Higher education	Ref.	Ref.	Ref.
**Parental behavioural control**		0.119 [0.117, 0.121]	0.029* [0.028, 0.03]	0.149 [0.146, 0.152]
**Parental limit setting**		-0.398*** [-0.39, -0.406]	-0.022 [-0.022, -0.022]	-0.42*** [-0.412, -0.428]
**Peer connection**		-0.286*** [-0.28, -0.292]	-0.001 [-0.001, -0.001]	-0.287*** [-0.281, -0.293]
**Peer regulation**		0.134* [0.131, 0.137]	0.002 [0.002, 0.002]	0.134* [0.131, 0.137]
**Peer psychological control**		-0.155 [-0.152, -0.158]	-0.002 [-0.002, -0.002]	-0.156 [-0.153, -0.159]

**Community psychological control**		NA	0.001 [0.001, 0.001]	0.001 [0.001, 0.001]
**Community disorganization**		NA	-0.001 [-0.001, -0.001]	-0.001 [-0.001, -0.001]
**Teacher's concern**		0.069*** [0.068, 0.07]	-0.001 [-0.001, -0.001]	0.068 [0.067, 0.069]

Discussion

The present study showed that adolescents' knowledge of pubertal changes is influenced significantly by various individual level, family level, and community-level factors. Sex, age, year of schooling, and academic performance were the individual level of factors that displayed a significant effect on adolescent's knowledge of pubertal changes. This current study revealed knowledge of pubertal changes positively influenced by age and years of schooling. A similar type of positive impact of age and year of schooling is also founded by Uddin and Choudhury [21]. An increase in the year of schooling increases knowledge more than the increase in age. An interesting finding of our analysis showed that female adolescents had more knowledge than the male. In a previous study on young people of Bangladesh illustrate that male adolescent had poor knowledge than the female [22]. Separate studies from India on school-going adolescents [23] and Portugal on college students [24] demonstrated that female adolescents had significantly higher knowledge than males. At the same time, religion plays an important role in the knowledge of pubertal changes. Muslim students had lower pubertal change knowledge than students of other religions. A previous study found that Hindu students had 1.76 times more likely to have better communication with mothers regarding SRH [5].

Among family-level factors, Mother's education shows a positive impact on adolescent's pubertal knowledge. These findings are also consistent with the study of Uddin and Choudhury [21] on adolescent girls in rural areas in Bangladesh and another study in India [5]. This is because daughters also have a trustworthy relationship with mother as a primary source of SRH information [5]. Though mothers are uncomfortable discussing SRH-related problems in our social structure, but educated mothers do not hesitate to talk about SRH issues [25]. While parents imposing limits on adolescents directly and indirectly reduce adolescents' pubertal knowledge. This finding depicts our country's tradition, where parents think that pubertal changes are a natural human development phenomenon that should need to remain secret [26]. However, parental behavioural control showed an indirect positive effect on

adolescent's knowledge on pubertal changes. This indicates that parents by imposing restrictions on adolescents' behaviour increase their knowledge of pubertal changes through the mediating variable of year of schooling and academic performance.

Peers and community are two important social components that have a great impact on adolescent functioning. This study found that both peer connection reduces adolescents' pubertal knowledge. Despite being a major source of pubertal and reproductive health knowledge, peer connection reduces adolescent's pubertal knowledge. This contradiction supports the previous finding of Das and Roy that peer almost always supplies incomplete and fantasy-oriented sexual and reproductive knowledge [16]. This may be the reason why peer connection reduces adolescent's pubertal knowledge instead of increasing. On the other hand, peer regulation showed a direct positive impact on adolescents' pubertal knowledge. Other important elements of society. Adolescents spend a significant portion of the time of their daily life passed in school. Therefore, school-level factors are essential for shaping their knowledge. Among other school-level factors, students' academic performance negative impact on adolescent's knowledge on puberty. On the other hand, the teacher's concern demonstrates an overall positive impact on adolescent's pubertal knowledge. Though teacher concern's direct effect is positive, the indirect effect of teacher's concern is negative. This evidence coincided with other separate previous studies and observed that when teachers exhibit care towards students, it motivates them to learn and eliminate and correct disruptive behaviour [27, 28].

Conclusion and Recommendations

In summary, we conclude that intervention, sex, years of schooling, age, parental limit-setting, peer concern, and peer regulation were the significant determinants on adolescent's knowledge of pubertal changes. The intervention that is provided sufficiently increased adolescent's knowledge on puberty. Based on this study it can be said that the content on Adolescent sexual and reproductive health of class 6 to 10 curricula, will enhance adolescent's knowledge significantly if intervention is provided. Therefore, based on the information that is obtained in this study it is recommended that intervention need to apply all over Bangladesh to make school-based sexual and reproductive health effects as well as to ensure adolescents safe and healthy sexual and reproductive health.

Acknowledgments

The authors are gratefully thankful to the department of statistics Jagannath University and the principal investigators of the project "Study on Effectiveness of Adolescent

Reproductive Health Service on Reducing Incidence of Early Marriage and Pregnancy" for the data.

Author Contributions

Conception and design of study: M Sheikh Giash Uddin and Md Injamul Haq Methun
Acquisition of data and methods: M Sheikh Giash Uddin and Md Injamul Haq Methun
Analysis of data: Md Injamul Haq Methun, Md. Ismail Hossain, and Md. Jakaria Habib
Drafting the manuscript: Md Injamul Haq Methun, Iqramul Haq, and Ahmed Abdus Saleh Saleheen
Revising the manuscript critically: Md Injamul Haq Methun, Iqramul Haq, and Md. Ismail Hossain

References

1. Deshmukh VR, Kulkarni AA, Apte SS. Knowledge and Attitude about Growing up Changes: An Intervention Study. Pediatric Oncall. 2014;11(3).
2. Sandhya P, Bimala P. Awareness and Attitude on Pubertal Changes among Community Adolescents. International Journal of Caring Sciences. 2017;10(2):1255–64.
3. Casey BJ, Duhoux S, Cohen MM. Adolescence: What Do Transmission, Transition, and Translation Have to Do with It? Neuron. 2010;67(5):749–60.
4. Schulz KM, Molenda-Figueira HA, Sisk CL. Back to the future: The Organizational–activational Hypothesis Adapted to Puberty and Adolescence. Hormones and Behavior. 2009;55(5):597–604.
5. Zakaria M, Xu J, Karim F, Cheng F. Reproductive Health Communication between Mother and Adolescent Daughter in Bangladesh: a cross-sectional Study. Reproductive Health. 2019;16(1).
6. Wheeler MD. Physical Changes of Puberty. Endocrinology and Metabolism Clinics of North America. 1991;20(1):1–14.
7. Biro FM, Greenspan LC, Galvez MP. Puberty in Girls of the 21st Century. Journal of Pediatric and Adolescent Gynecology. 2012;25(5):289–94.
8. Özdemir A, Utkualp N, Palloş A. Physical and Psychosocial Effects of the Changes in Adolescence Period. International Journal of Caring Sciences. 2016;9(2):717–23.
9. Ghai OP, Gupta P, Paul VK. Ghai Essential Pediatrics. 6th ed. 2019.
10. Blakemore S-J, Burnett S, Dahl RE. The Role of Puberty in the Developing Adolescent Brain. Human Brain Mapping. 2010;31(6):926–33.
11. Berk LE. Development through the Lifespan. 6th ed. New York: Pearson; 2014.

12. Reena M. Psychological Changes during Puberty -Adolescent School Girls. Universal Journal of Psychology. 2015;3(3):65–8.
13. Yasmin R, Rob U, Hena IA, Das TR, Ahmed FU. Increasing Access to Safe Menstrual Regulation Services in Bangladesh by Offering Medical Menstrual Regulation. Reproductive Health Matters. 2014;22(44 suppl 1):67–74.
14. Ministry of Health and Family Welfare, Government of the People's Republic of Bangladesh. NATIONAL STRATEGY FOR Ministry of Health and Family Welfare Government of the People’s Republic of Bangladesh. Dhaka, Bangladesh: MCH Services Unit; 2017.
15. Kumari N, Mehta DrSuheli. Knowledge regarding Pubertal Changes among Adolescent girls: A Cross Sectional Study. International Journal of Basic and Applied Research. 2019;9(5):356–64.
16. Das AK, Roy S. Unheard Narratives of Sexual and Reproductive Health rights (SRHR) of Adolescent Girls of the Holy Cross college, Dhaka, Bangladesh. IOSR Journal of Humanities and Social Science. 2016;21(2):1–8.
17. Ainul S, Bajracharya A, Reichenbach L. Adolescents in Bangladesh Programmatic Approaches to Sexual and Reproductive Health Education and Services. Dhaka: Population Council; 2016.
18. Urie Bronfenbrenner. Ecology of Human Development: Experiments by Nature and Design. Cambridge: Harvard University Press; 1979.
19. Amoateng AY, Kalule-Sabiti I. Biosocial Correlates of Age at First Sexual Intercourse: The Case of Grade 9 And Grade 11 Pupils in The North West Province of South Africa. Journal of Biosocial Science. 2014;48(1):20–36.
20. Rosseel Y. lavaan: An R Package for Structural Equation Modelling. Journal of Statistical Software. 2012;48(2).
21. Uddin MdJ, Choudhury AM. Reproductive Health Awareness among Adolescent Girls in Rural Bangladesh. Asia Pacific Journal of Public Health. 2008;20(2):117–28.
22. Hossain SM. Knowledge on Sexual and Reproductive Health among Young People in a Selected Community. Journal of Gynecology and Womens Health. 2016;1(3).
23. Deshmukh DD, Chaniana SS. Knowledge about Sexual and Reproductive Health in Adolescent School-Going Children of 8th, 9th, and 10th Standards. Journal of Psychosexual Health. 2020;2(1):56–62.
24. Santos MJ, Ferreira E, Ferreira M. Knowledge of and Attitudes toward Sexual and Reproductive Health among College Students. Aten Primaria. 2016;48((Espec Cong

1)):188–94.
25. Kumar A, Srivastava K. Cultural and Social Practices regarding Menstruation among Adolescent Girls. Social Work in Public Health. 2011;26(6):594–604.
26. Poojary CD, John D, Babu C, D'souza RP, Shetty AP. Pubertal changes: Knowledge among School Aged Girls. American International Journal of Research in Humanities, Arts and Social Sciences. 2015;12(1):51–3.
27. Uslu F, Gizir S. School Belonging of Adolescents: The Role of Teacher–Student Relationships, Peer Relationships and Family Involvement. Educational Sciences: Theory & Practice. 2016;17(1).
28. Ma X. Sense of Belonging to School: Can Schools Make a Difference? The Journal of Educational Research. 2003;96(6):340–9.

Click here to access/download;Figure;Figure 1.docx

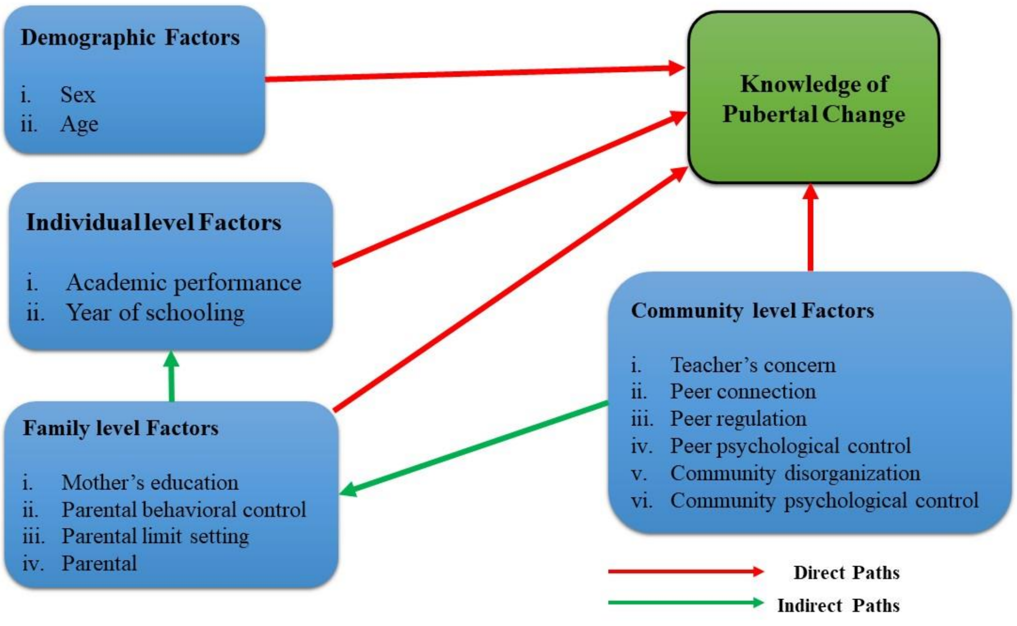

Figure 1. Conceptual Frame work
