# Supplementary material for: Biosocial correlates of adolescent’s knowledge on pubertal changes in rural Bangladesh: A structural equation model
Source: PLoS One. 2022 Mar 22;17(3):e0264515. doi: 10.1371/journal.pone.0264515 (PMC8939805; doi:10.1371/journal.pone.0264515)
Supplement: Attachment — Submitted filename: manuscript paper review.docx [file pone.0264515.s002.docx]

**Manuscript review**

This study is very important and has useful insights about factors that relate with adolescent's knowledge on pubertal changes.

**Major comments**

| **Section** | **Comment** |
| --- | --- |
| **Title** | - The study focus based on the title is not similar with the aim of the study - The independent variable **(Sexual and Reproductive Health Education)** was not included in the questionnaire and in data analysis - The aim of the study as shown in the abstract was to **“identify factors that are associated with puberty knowledge among school-going rural adolescents in Bangladesh”** - Suggestion: The title should be revised |
| **Introduction** | - Well written - Initiatives mentioned in the introduction regarding programs initiated by the government and NGOs were not included in the questionnaire to measure impact of Sexual and Reproductive Health Education intervention - Sexual and Reproductive Health Education courses or training not included in the Conceptual Framework |
| **Method** | - Design is appropriate and participant selection procedure was included - The sample size was adequate to perform the analysis with 16 variables. - Variables well defined - The study should indicate if the questionnaire was pretested |
| **Analysis** | - The model data and description of significance is clear |
| **Conclusion and recommendation** | - The conclusion and recommendation is weak - In the conclusion, **intervention** was not included as a determinant - The statement **(… study provides insights into how school-based sexual health education can be made more effective to increase adolescent's knowledge about pubertal changes in rural Bangladesh**” does not align with the factors analyzed in this study - Major factor directly and significantly influencing the knowledge of puberty changes should be used in the conclusion and recommendation |
| **General** | - Minor English edits needed - Accept the manuscript with minor edits |
