# Supplementary material for: Biosocial correlates of adolescent’s knowledge on pubertal changes in rural Bangladesh: A structural equation model
Source: PLoS One. 2022 Mar 22;17(3):e0264515. doi: 10.1371/journal.pone.0264515 (PMC8939805; doi:10.1371/journal.pone.0264515)
Supplement: Attachment — Submitted filename: Response to Reviewers.docx [file pone.0264515.s003.docx]

**Responses to the remarks of editors and reviewers:**

| Response to the editors remarks. | |
| --- | --- |
| 1. Please ensure that your manuscript meets PLOS ONE's style requirements, including those for file naming. The PLOS ONE style templates can be found at  <https://journals.plos.org/plosone/s/file?id=wjVg/PLOSOne_formatting_sample_main_body.pdf> and  <https://journals.plos.org/plosone/s/file?id=ba62/PLOSOne_formatting_sample_title_authors_affiliations.pdf> | We have followed the instruction of PLOS ONE style. |
| 2. You indicated that you had ethical approval for your study. In your Methods section, please ensure you have also stated whether you obtained consent from parents or guardians of the minors included in the study or whether the research ethics committee or IRB specifically waived the need for their consent. | In method section we have clearly stated the consent which are taken before data collections.  [line 162 -163] |
| 3. PLOS requires an ORCID iD for the corresponding author in Editorial Manager on papers submitted after December 6th, 2016. Please ensure that you have an ORCID iD and that it is validated in Editorial Manager. To do this, go to ‘Update my Information’ (in the upper left-hand corner of the main menu), and click on the Fetch/Validate link next to the ORCID field. This will take you to the ORCID site and allow you to create a new iD or authenticate a pre-existing iD in Editorial Manager. Please see the following video for instructions on linking an ORCID iD to your Editorial Manager account: <https://www.youtube.com/watch?v=_xcclfuvtxQ> | ORCID id have been attached. |
| 4. Please ensure that you refer to Figure 1 in your text as, if accepted, production will need this reference to link the reader to the figure. | We have referred Figure 1 in the text [line -126] |
| 5. Please include a copy of Table 5 which you refer to in your text on page 11. | There was mistake, actually it was Table -4. We have amended the Table number. |

| **Responses to the reviewers remarks :** | |
| --- | --- |
| **Reviewer-1** | |
| The manuscript is technically sound and the statistical analysis has been performed appropriately but they have mentioned that data cannot be shared publicly because of the restriction of data authority. However, they can add data course link. The manuscript needs major revisions as sentences are not very clear. | We revised the manuscript. |
| **Reviewer-2** | |
| 1. The justification given as a rational for the study- "No specific research has been done so far on how parents and our society hinder adolescent's knowledge of pubertal changes."- seems a bit odd. There are several published studies done in rural Bangladesh and several Asian countries and probably specific research might not be done in the specific study area in the past, and it should not be generalized. | We have amended the justification. [Line: 104-107] |
| 2. I am not clear if there was a parental consent since the mean age in this study was 14.67 Years. Did the researchers used other mechanisms to ensure parental consent? | Parental consent was taken. We have made an attempt to clarify this issue in line 161-163. |
| 3. One of the variables relate to Student's academic performance by directly asking the student. It might have been better if the actual performance forms the academic year was assessed. | Since adolescents were interviewed from both household and school, it is difficult to assess academic performance forms the academic year performance. Therefore, Student's academic performance by directly asking the student. [Line: 234 – 237] |
| 4. The discussion tried to compare results with other studies but the why part of the results are not included in the discussion part. As an example, the female adolescent's understanding of puberty was higher that male, which might be related to having the onset menses (Menstrual period) during that age under study. So such justifications are important to give the reader in knowing what reasons are linked to the findings of the study. | We have revised the discussion section of the manuscript. |
| 5. I don't see any limitation of the study included to justify some of the drawbacks which might be very useful for the reader to understand those factors which hinder the researchers. | We have added limitations of the study. |
| 6. Part of the recommendation can include possible future studies to be done based on the findings of the study. | We have revised the recommendation section. |
| 7. The title of the study relates to impact of school based SRH education, but this is not clearly seen in the study and it might be good to modify the title to represent the study methodology and findings. | **We have modified the title as “****Biosocial correlates of adolescent’s knowledge on pubertal changes in rural Bangladesh: a structural Equation model”** |
| **Reviewer-3** | |
| 1. The paper "Impact of School-based Sexual and Reproductive Health Education among Secondary Student’s Knowledge on Pubertal Changes in Rural Bangladesh" undertakes an interesting topic. It seems like the study has certain potential. The results are interesting and it seems that authors were able to put their findings within the general literature context. However, the study lacks considerably in terms of readability and academic rigor of presentation. Below I present my more specific remarks but I encourage you to thoroughly proof read the manuscript. | We have revised our manuscript to improve the readability of the manuscript. |
| **Abstract**  2. "parental limit setting" should be explained more thoroughly. | We have replaced "parental limit setting" with “parental limit setting on daily activities” in the result of the abstract. [ line 22-23]. Moreover, the explanation of “parental limit setting is given in contextual factors under methodology [line: 197 to 203] |
| 3. Please refrain from using causal language (e.g., influencing) in your cross-sectional study. | We have revised our manuscript and replace the causal language. [line no. 53,55] |
| 4. Your claim that some of your variables were "influencing" knowledge on puberty both directly and indirectly suggests that you were using some mediation analysis. The article would benefit if you were more explicit about that fact and also, if you could state the reasons for running mediation analysis. | We have included the reasons of running mediation analysis in the methodology section of the abstract [line 40 to 43] |
| 5. Instead of simple conclusions, you could provide some policy implications to improve knowledge about pubertal changes in Bangladesh. | We have revised the conclusion section of the manuscript. |
| **Introduction**  6. What do you mean by "To make healthy dults from childhood, ..."? This sentence is very unclear. I would suggest to omit first three sentences in your introduction. | We have omitted the first three line of the introduction. |
| 7. You claim that "society is responsible for ensuring informed puberty" but probably even more responsibility lies in the family and in the schooling system. | Replacing Society with family.  [line: 87] |
| 8. Please refrain from using "condition" in relation to puberty. It is rather a developmental stage. | We have made an amendment to refrain from using causal language. |
| 9. Again, in stating your objectives please refrain from causal language (e.g., affect). | We have made an amendment to refrain from using causal language |
| **10. Conceptual Framework**  It would be worth describing which elements of bioecological model of Bronfenbrenner were included in your framework and what is the conceptual novelty of your approach. In the current version it is not clear. | We have elaborately described the Conceptual framework section. |
| 11.The section on data is not clear. Individuals between 10 and 18 or 10 and 24 were sampled? 3014 or 3000 were surveyed? 1504+1509 is not equal to 3014. "VI to IX" or "6-9"? | Individual between 10-24 were sampled. 3013 were surveyed. We have revised our manuscript with VI-IX. |
| 12. It is not clear while "outcome variable" is under the heading of "study population". Please explain. | The manuscript is rewritten to increase the clarity of the manuscript. |
| 13. Knowledge of pubertal changes scale should be somehow described. Questionnaire should be put in the appendix. If the scale is used, its psychometric properties should be checked - at least reliability of the scale. | We included the reliability. [ line: 241-247] |
| 14. The scales used in the study (PBC, PLS, CD, CPC, PeC, PePC, PeR, TC) should have their psychometric properties checked. Reader should also have access to detailed wording of the items comprising each scale. | We included the reliability. [ line: 241-247] |
| **Statistical Analysis**  15. Again, please refrain from causal language. | We have made an amendment to refrain from using causal language. |
| 16. Please elaborate more on specification and estimation of your structural equation model. | The statistical analysis section have been rewritten with an elaboration of specification and estimation of structural equation model. [ Line: 256-261] |
| 17. Why you have not decided to use latent constructs in your model? Your data is perfectly fit to such an approach. | We have included the explanation of not using latent construct between line 176 to 183. |
| **Results**  18. In the analysis of contextual factors why do you use median when for the socio-economic characteristics you were using mean? | For continuous socio-economic characteristics, mean have been used and for ordinal contextual factors median have been used. We have included the explanation between line 2513 to 253. |
| 19.Table 3 contains variable KAP, it is not clear what it means? | We made an attempt to clarify the meaning the variable |
| 20. Lack of reference categories for variables like "Gender", "Religion" precludes understanding of the results in Table 3. | We made an amendment of Table 3 with the reference categories. |
| 21. When presenting indirect effects in Table 4, I would suggest to include mediating variable. It is difficult to understand to which indirect paths the results refer. | We have included a column of mediator variables in Table 4. |
| **22. Discussion**  What is the meaning of SRH? The acronym has not been introduced. | We have amended the discussion section. [line 374] |
| **Reviewer-4** | |
| Question 1: The Sexual and Reproductive Health Education variable was not included in the questionnaire and in data analysis. The conclusion is based on this intervention variable which was not part of the analysis | We have revised our manuscript. |
| Question 3: Some restriction applied on data availability |  |
