# Supplementary material for: Biosocial correlates of adolescent’s knowledge on pubertal changes in rural Bangladesh: A structural equation model
Source: PLoS One. 2022 Mar 22;17(3):e0264515. doi: 10.1371/journal.pone.0264515 (PMC8939805; doi:10.1371/journal.pone.0264515)
Supplement: Attachment — Submitted filename: Response to Reviewers.docx [file pone.0264515.s004.docx]

**Responses to the remarks of reviewers:**

1.I would suggest revising the statements in lines 175-177 "Latent construct approach is the most suitable approach for measuring. However, to construct a latent variable it is essential to remove observed variables if the correlations between variables is very high or is not high-enough." It is not clear what is the intention behind the statement.

Response: We have revised the lines 175-177.

2.Could you provide a bit more elaborate description of the Kalule-Sabiti method. 2-3 additional sentences could help better understand the approach.

Response: We have a brief description of Kalule-Sabiti method in lines 191 to 196.

3. In line 287 you mention that "The structural equation model was over-identified," but it is unclear what kind of specification you suggest for the model. I would suggest that you draw your model with all potential covariates to show your starting specification.

Response: We have explained the specification procedure in lines 293 – 297.

**Minor:**

"pubertal period" sounds a bit artificial - please change this.

Response: In line 78 and 62, we replace “pubertal period” with “puberty”.

In line 78 changes with puberty. In line 62 changes with puberty

Line 84: should be "prevent"
Response: Replaced.

Line 90 "think" sounds a bit strange in this context.

Response: In line 90, we replace “think” with “Parents left”.

Line 91: Maybe instead of "mass media", current teenagers refer more to social media.

Response: Yes, we have amended the line.

Line 101: Replace "find out" with "identify"

Response: Replaced with identify

Line 130: Replace "carried out" with "provided"

Response: Replaced with “provided”.

Line 139: Revise the sentence.

Figure 1: "framework" instead of "frame work"

Response: Replaced with “framework”

Line 222-223: The inverted commas system is not clear.

Response: The inverted commas system is rearranged.

Line 242: "using" instead of "sing"

Response: Relaced with “using”.

Line 163: Shouldn't it be "approved by" instead of "taken from"?

Response: Replaced with “approved by”.
