# Supplementary material for: Biosocial correlates of adolescent’s knowledge on pubertal changes in rural Bangladesh: A structural equation model
Source: PLoS One. 2022 Mar 22;17(3):e0264515. doi: 10.1371/journal.pone.0264515 (PMC8939805; doi:10.1371/journal.pone.0264515)
Supplement: Attachment — Submitted filename: Response to Reviewers.docx [file pone.0264515.s005.docx]

Dear Ammal mokhtar Metwally,

Thank you for giving us the opportunity to submit a revised draft of my manuscript titled “Biosocial correlates of adolescent’s knowledge on pubertal changes in rural Bangladesh: a structural Equation model” to *Plos One.* We appreciate the time and effort that you and the reviewers have dedicated to providing your valuable feedback on my manuscript. We are grateful to the reviewers for their insightful comments on my paper. We have been able to incorporate changes to reflect most of the suggestions provided by the reviewers. Here is a point-by-point response to the reviewers’ comments and concerns.

**Responses to the remarks of reviewers:**

1.Please be more specific in your statement "parents and peers are negatively associated with adolescent’s understanding of pubertal changes". It is not clear what you mean.

Response: We have revised the line. (Line 50 and 51)

2. Line 68 "in girls" seems redundant.

Response: line 60: we deleted "in girls".

3. Line 77 - "recognize" is not a correct word here (maybe "accept" or "endorse" would be better)

Response: line 69 – “recognize’ replaced with “accept”.

4. Line 82 - "expose" needs a pronoun here

Response: line 74- We have added pronoun.

5. Line 84 - "prevents" is not correct

Response: line 76 – we replaced “prevents”.

6. Lines 92-94 - the sentences are not clear

Response: line 84 – 88 – we have amended the sentences.

7. "upzila"'s meaning should be explained

Response: We added the meaning of “upazila” in line 135.

8. Line 182 - still not clear what you mean when you say "using above approach"

Response: line 176-177 – We have revised the sentence.

9. Line 186 - instead of saying "independent variables" I would suggest "control variables"

Response: line 179 - "independent variables" replaced with "control variables".

10. Please read carefully your description of "Contextual factors". It requires a through proof reading.

Response: We have revised contextual factors.

11. Line 281 - what do you mean by saying "parental limit setting ranging from 1 to 4 and the upper limit for the remaining variables is 5"?

Response: The line is revised. (Line 277-278.)

12. Line 300 - what is the "model fit test statistic" that you are referring to?

Response: We have included in line 296.

13. The values of model fit (GFI, RMSEA) are rather acceptable and not good.

Response: We have made amendment the statement. (Line 297 -299).

14. In notes for Table 3, please describe the abbreviations used.

Response: We have added the abbreviations.

15. Table 3 - please comment whether the coefficients are standardized or unstandardized.

Response: standardized

16. Please avoid causal language in your analysis. Your sample is cross-sectional and words like "affected" should not be used. Replace with "correlated", for example. Check the whole manuscript and eliminate causal language.

Response: we made an amendment

17. I see discrepancies between the coefficients presented in Table 3 and in the description provided in "Direct effects, indirect effects, and total effect". Please correct it.

Response: we revised the manuscript.

Sincerely,

Md. Injamul Haq Methun

Lecturer

Tejgaon College

Dhaka, Bangaldesh.
